# Fungal Light-Oxygen-Voltage Domains for Optogenetic Control of Gene Expression and Flocculation in Yeast

**DOI:** 10.1128/mBio.00626-18

**Published:** 2018-07-31

**Authors:** Francisco Salinas, Vicente Rojas, Verónica Delgado, Javiera López, Eduardo Agosin, Luis F. Larrondo

**Affiliations:** a Departamento de Genética Molecular y Microbiología, Facultad de Ciencias Biológicas, Pontificia Universidad Católica de Chile, Santiago, Chile; b Millennium Institute for Integrative Systems and Synthetic Biology (MIISSB), Santiago, Chile; c Centro de Estudios en Ciencia y Tecnología de los Alimentos (CECTA), Universidad de Santiago de Chile (USACH), Santiago, Chile; d Department of Chemical and Bioprocess Engineering, School of Engineering, Pontificia Universidad Católica de Chile, Santiago, Chile; University of Melbourne

**Keywords:** LOV domain, filamentous fungi, flocculation, heterologous gene expression, optogenetics, yeasts

## Abstract

Optogenetic switches permit accurate control of gene expression upon light stimulation. These synthetic switches have become a powerful tool for gene regulation, allowing modulation of customized phenotypes, overcoming the obstacles of chemical inducers, and replacing their use by an inexpensive resource: light. In this work, we implemented FUN-LOV, an optogenetic switch based on the photon-regulated interaction of WC-1 and VVD, two LOV (light-oxygen-voltage) blue-light photoreceptors from the fungus Neurospora crassa. When tested in yeast, FUN-LOV yields light-controlled gene expression with exquisite temporal resolution and a broad dynamic range of over 1,300-fold, as measured by a luciferase reporter. We also tested the FUN-LOV switch for heterologous protein expression in Saccharomyces cerevisiae, where Western blot analysis confirmed strong induction upon light stimulation, surpassing by 2.5 times the levels achieved with a classic *GAL4*/galactose chemical-inducible system. Additionally, we utilized FUN-LOV to control the ability of yeast cells to flocculate. Light-controlled expression of the flocculin-encoding gene *FLO1*, by the FUN-LOV switch, yielded flocculation in light (FIL), whereas the light-controlled expression of the corepressor *TUP1* provided flocculation in darkness (FID). Altogether, the results reveal the potential of the FUN-LOV optogenetic switch to control two biotechnologically relevant phenotypes such as heterologous protein expression and flocculation, paving the road for the engineering of new yeast strains for industrial applications. Importantly, FUN-LOV’s ability to accurately manipulate gene expression, with a high temporal dynamic range, can be exploited in the analysis of diverse biological processes in various organisms.

## IMPORTANCE

Optogenetic switches are molecular devices which allow the control of different cellular processes by light, such as gene expression, providing a versatile alternative to chemical inducers. Here, we report a novel optogenetic switch (FUN-LOV) based on the LOV domain interaction of two blue-light photoreceptors (WC-1 and VVD) from the fungus N. crassa. In yeast cells, FUN-LOV allowed tight regulation of gene expression, with low background in darkness and a highly dynamic and potent control by light. We used FUN-LOV to optogenetically manipulate, in yeast, two biotechnologically relevant phenotypes, heterologous protein expression and flocculation, resulting in strains with potential industrial applications. Importantly, FUN-LOV can be implemented in diverse biological platforms to orthogonally control a multitude of cellular processes.

## INTRODUCTION

Control of gene expression is an important tool for basic and applied research in biology. Chemical inducers, such as isopropyl-β-d-thiogalactopyranoside (IPTG), methanol, or galactose, have been profusely employed for inducible gene regulation despite their obvious limitations, including potential interference with metabolic processes, difficulty in removal from the culture medium once added, and insufficient temporal and spatial/dose resolution (1). In addition, the cost of chemical inducers can restrict their uses in some industrial applications, whereas temperature induction or constitutive gene expression is chosen based on cost efficiency regardless of its far-from-optimal characteristics (2). Light constitutes a promising alternative for the control of gene expression, considering its low cost, reduced toxic effects, adjustable levels, and high temporal and spatial resolution (3). In several organisms, light readily controls different processes, including gene expression, through photoreceptors with specialized domains which, under light stimulation, undergo a conformational change, passing to an active state (4). Such photoreceptors have allowed the definition of basic building blocks from which to develop optogenetic switches, which can be engineered into synthetic light-controlled orthogonal transcription factors (5). During the past several years, a nascent repertoire of optogenetic switches responding to light of different wavelengths, and assembled in different platforms, has become available (6–11).

Fungal photoreceptors have been an underexploited source of biological parts for the implementation of optogenetic switches. The Neurospora crassa blue-light photoreceptor VIVID (VVD) has the capacity to self-dimerize upon light stimulation through its light-oxygen-voltage (LOV) domain (12). This feature of VVD already led to the development of the optogenetic system named “LightOn,” which was successfully utilized for light-controlled expression of transgenes in mice and mammalian cells (13, 14). Notably, in *Neurospora*, VVD also interacts with the blue-light photoreceptor White Collar 1 (WC-1), through WC-1’s LOV domain, allowing N. crassa to photoadapt in the presence of continuous illumination (15–17).

The budding yeast Saccharomyces cerevisiae ranks among the most relevant and versatile microorganisms for biotechnology. The absence of photoreceptors in the yeast genome (18) has fueled the implementation of different optogenetic switches in this biological chassis, allowing the orthogonal control of diverse cellular processes by light (19–21). However, despite their obvious advantages, the use of optogenetic switches to control biotechnologically relevant phenotypes in yeast has been seldom implemented (22). For example, optogenetic switches for heterologous protein expression in yeast would replace the addition of chemical inducers, reducing the cost of industrial-scale bioprocesses, providing also a dynamic control of gene expression and the effective temporal control of the on and off states. Another biotechnologically relevant operation in yeast fermentation is flocculation, which allows a fast, accessible, and efficient way to remove remaining yeast cells after fermentation processes (23). Flocculation is controlled by the *FLO* genes, a family of subtelomeric genes which trigger cell aggregation upon nutrient starvation or under environmental stress conditions (24, 25). These features position flocculation as an ideal and biotechnologically relevant target for optogenetic control.

In this work, we present as proof of concept a new optogenetic switch that provides an ample dynamic range of expression, with low background in the off (dark) state. Its implementation in yeast provides evidence of tight regulation of gene expression by blue light and the control of biotechnologically relevant features and phenotypes, such as heterologous protein expression and flocculation.

## RESULTS

### FUN-LOV provides a dynamic range of gene expression.

**FIG 1:**
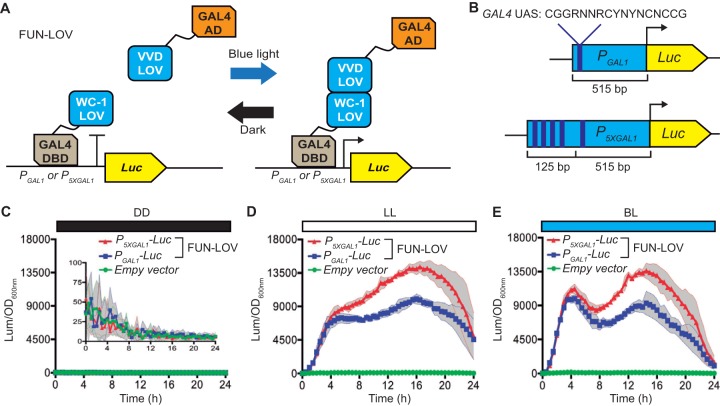
The FUN-LOV switch allows high levels of gene expression by light. (A) FUN-LOV is based on the light-controlled interaction of the LOV (light-oxygen-voltage) domains of WC-1 and VVD photoreceptors which are tethered to the Gal4 DNA binding domain (DBD) and Gal4 activation domain (AD), respectively. (B) Design of the synthetic promoter *P*_*5XGAL1*_, which contains four additional Gal4-UAS sequences upstream of the *GAL1* (*P*_*GAL1*_) promoter sequence. Both promoters are controlling luciferase (Luc) expression, which serves as a reporter of FUN-LOV activity. (C to E) Luciferase expression under constant darkness (DD) (C), constant white light (LL) (D), and constant blue light (BL) (E). In panels C to E, standard deviations are represented as shadowed regions.

The new optogenetic switch, named FUN-LOV, was developed based on the pairing of WC-1 and VVD LOV domains, an interaction known to occur as part of the N. crassa photoadaptation process (17). The configuration of the FUN-LOV switch follows a yeast two-hybrid system design logic, where the LOV domain of WC-1 is bound to a *Gal4* DNA binding domain (GAL4-DBD) and the full-length VVD (which contains a LOV domain) is tethered to the *Gal4* transactivation domain (GAL4-AD). Thus, upon the light-stimulated interaction of this LOV domain pair, a functional transcription factor is reconstituted, activating transcription as evidenced by a destabilized firefly luciferase reporter (*Luc*) under the control of the *GAL1* promoter (*P*_*GAL1*_) (Fig. 1A). Additionally, with the aim to increase gene expression induction even further, we designed a synthetic version of the *GAL1* promoter (*P*_*5XGAL1*_), which included four additional *GAL4-UAS* DNA binding site sequences (Fig. 1B).

**FIG 2:**
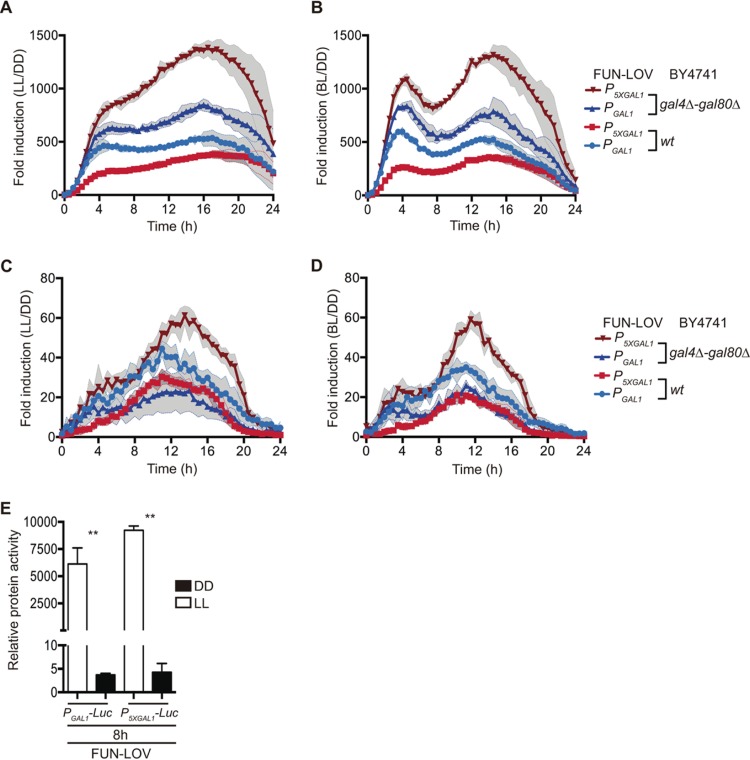
Fold induction achieved by the FUN-LOV system. Luciferase expression in constant white light (LL) (A and C) or constant blue light (BL) (B and D), with respect to the average background expression in constant darkness (DD). (A and B) Data from episomal luciferase reporters under the control of two different promoters (*P*_*GAL1*_ and *P*_*5XGAL1*_) and assayed in two different genetic backgrounds (BY4741 and BY4741 *gal4Δ-gal80Δ*). (C and D) Data from luciferase reporters inserted at the *GAL3* locus under the control of *P*_*GAL1*_ and *P*_*5XGAL1*_ promoters, in two different genetic backgrounds (BY4741 wt and BY4741 *gal4Δ-gal80Δ*). (E) Luciferase activity from protein extracts of cells harvested at the 8-h time point. The double asterisk represents a significant statistical difference between LL and DD conditions (*t* test, *P* < 0.01). In panels A to D, the standard deviations are represented as shadowed regions.

FUN-LOV revealed high levels of luciferase expression under constant white-light (LL) or constant blue-light (BL) conditions, with notably low levels of background expression in constant darkness (DD) (Fig. 1C, D, and E; see also Fig. S1 and S2 in the supplemental material). In our hands, the expression levels achieved by the FUN-LOV switch were superior to those obtained by applying classical galactose induction (Fig. S2). Furthermore, the maximum luciferase expression levels of the system were 1,218-fold induction for white light and 1,316-fold induction for blue light, utilizing the synthetic *P*_*5XGAL1*_ promoter (Fig. 2A, B, C, and D). The FUN-LOV system yielded similar results employing two different yeast genetic backgrounds (including the absence of endogenous Gal4/Gal80 proteins) and displayed robust luciferase expression either when maintained episomally or when integrated in the genome (Fig. 2, S1, and S2). As luminescence was being measured directly from living cells, we wanted to confirm that luciferase levels from protein extracts would reflect the same range of induction. Therefore, luciferase activity was measured in extracts from cells grown under constant light (LL) or darkness (DD) for 8 h, ratifying the strength of the FUN-LOV switch and revealing even higher levels of induction (~2,500-fold, Fig. 2E). Thus, the FUN-LOV switch provides remarkably high levels of gene expression, as measured by a luciferase reporter, under constant illumination with low background expression in darkness.

10.1128/mBio.00626-18.1 FIG S1  FUN-LOV control of luciferase expression. Bioluminescence was monitored and normalized by OD_600_, as cultures were grown in constant darkness (DD), constant white light (LL), or constant blue light (BL), respectively. (A to F) Components of the FUN-LOV system and the reporter construct (*P*_*GAL1*_-luciferase or *P*_*5XGAL1*_-luciferase) were maintained episomally in a BY4741 wild-type strain (A to C) or in a BY4741 *gal4Δ-gal80Δ* strain (D to F). (G to L) BY4741 (G to I) or BY4741 *gal4Δ-gal80Δ-*derived strains (J to L), containing the FUN-LOV system episomally, but having the luciferase reporters integrated at the *GAL3* locus. In panels A to L, standard deviations are represented as shadowed regions. Download FIG S1, PDF file, 0.8 MB. Copyright © 2018 Salinas et al. 2018 Salinas et al. This content is distributed under the terms of the Creative Commons Attribution 4.0 International license.

10.1128/mBio.00626-18.2 FIG S2  Raw luciferase expression levels during active yeast growth. (A to C) The growth of yeast strains containing FUN-LOV and luciferase controlled by a *P*_*GAL1*_ or *P*_*5XGAL1*_ promoter was monitored as OD_60_ and bioluminescence levels, which were quantified in constant darkness (DD), constant white light (LL), or constant blue light (BL), respectively. (A to F) Components of the FUN-LOV switch and the reporter gene were maintained episomally in the BY4741 wild type (A to C) or BY4741 *gal4Δ-gal80Δ* strain (D to F). (G to L) Although the FUN-LOV switch was kept episomally, the luciferase reporter was chromosomally inserted at the *GAL3* locus in BY4741 (G to I) or in the BY4741 *gal4Δ-gal80Δ* (J to L) background. (M and N) In addition, the behavior of the episomal Luc reporter in a BY4741 background, depending on the endogenous Gal4p, was evaluated in glucose (M) or galactose (N). When indicated, raw data for luciferase expression were acquired from two different types of promoters, *P*_*GAL1*_ and *P*_*5XGAL1*_. In panels A to N, standard deviations are represented as shadowed regions. Download FIG S2, PDF file, 1.3 MB. Copyright © 2018 Salinas et al. 2018 Salinas et al. This content is distributed under the terms of the Creative Commons Attribution 4.0 International license.

**FIG 3:**
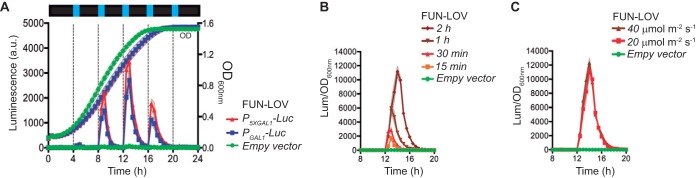
FUN-LOV allows dynamic control of gene expression. (A) Luciferase expression in yeast cells subjected to 30 min of blue-light pulses every 4 h. (B) Luciferase expression under blue-light pulses of different duration. (C) Luciferase expression using two different light intensities applied for 2 h. In panels B and C, data correspond to luciferase under the control of a *P*_*5XGAL1*_ promoter. In panels A to C, standard deviations are represented as shadowed regions.

The feasibility of operating the FUN-LOV system as an on/off switch with dynamic and temporal resolution was also assayed. The FUN-LOV system yielded a distinct dynamic and temporal control of luciferase expression during the yeast exponential growth phase (Fig. 3A and S3). The system exhibited a decay in the stationary phase, probably resulting from the low transcriptional activity of the promoter (*P*_*ADH1*_) controlling the expression of the FUN-LOV components, which appeared as a common feature of the constitutive promoters evaluated in this work (Fig. S4). This also suggests that the expression levels reached by the FUN-LOV system might be further incremented by expressing its components under stronger promoters, such as *P*_*TEF1*_ or *P*_*TDH3*_ (Fig. S4C and D), which in the future could further boost the relative levels of the FUN-LOV switch components, such as the GAL4-DBD moiety (Fig. S4E). We also found that longer exposure to a blue-light pulse increased the expression of the reporter gene for yeast cells growing in exponential phase. Thus, the system reached its maximum levels after 2 h of light exposure (Fig. 3B and S3E). However, an increase in the blue light-emitting diode (LED) light intensity (from 20 to 40 µmol m^−2^ s^−1^) did not further augment luciferase levels (Fig. 3C and S3F), probably reflecting a saturation of the system at 20 µmol m^−2^ s^−1^ based on the amount of existing photoreceptor molecules. Overall, the FUN-LOV system offers high expression levels of the luciferase reporter gene upon light, with great dynamic and temporal resolution, making it a suitable switch for the control of biotechnological phenotypes in yeast (26).

10.1128/mBio.00626-18.3 FIG S3  Tuneable and dynamic control of luciferase expression by the FUN-LOV switch. Bioluminescence was actively monitored as yeast cells were grown in constant darkness and 30-min blue-light pulses were applied as depicted (A to D). *P*_*GAL1*_-*Luc* or *P_5XGAL1_-Luc* reporters were maintained episomally in a BY4741 (A) or BY4741 *gal4Δ-gal80Δ* (B) background or inserted at the *GAL3* locus in BY4741 (C) or BY4741 *gal4Δ-gal80Δ* (D). The effect of the duration (E) or intensity (F) of a blue-light pulse was evaluated in a strain containing an episomally maintained *P_GAL1_-Luc* reporter. In panel F, the duration of the pulse was 2 h. In panels A to F, standard deviations are represented as shadowed regions. Download FIG S3, PDF file, 0.4 MB. Copyright © 2018 Salinas et al. 2018 Salinas et al. This content is distributed under the terms of the Creative Commons Attribution 4.0 International license.

10.1128/mBio.00626-18.4 FIG S4  Behavior of the constitutive promoter controlling the FUN-LOV components. (A) Expression of the FUN-LOV switch components (also Fig. 1A) is regulated by the *ADH1* promoter (*P*_*ADH1*_) and *ADH2* transcriptional terminator (*ADH2*_*ter*_). (B to D) The graphs depict luciferase expression regulated by the *P*_*ADH1*_ (B), *P*_*TEF1*_ (C), and *P*_*TDH3*_ (D) promoters, as well as the OD_600_. (E) *GAL4-DBD* expression was measured by real-time PCR (qPCR) in two different genetic backgrounds (BY4741 and BY4741 *gal4*Δ-*gal80*Δ) and with/without the FUN-LOV system. In panels B to D, standard deviations are represented as shadowed regions. Download FIG S4, PDF file, 0.3 MB. Copyright © 2018 Salinas et al. 2018 Salinas et al. This content is distributed under the terms of the Creative Commons Attribution 4.0 International license.

### FUN-LOV control of heterologous protein production.

**FIG 4:**
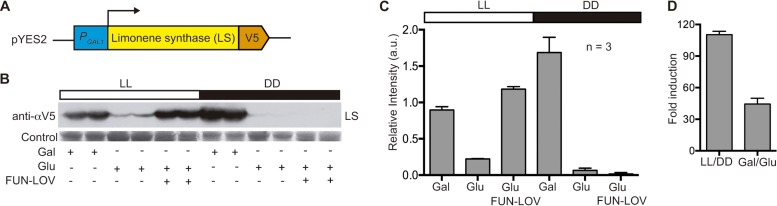
Heterologous protein expression controlled by the FUN-LOV switch. (A) The limonene synthase protein (LS) was expressed under the control of the *P*_*GAL1*_ promoter, including a C-terminal V5 tag for Western blot detection. (B and C) LS expression was analyzed by Western blotting (B) and normalized to membrane-stained proteins (C), after the yeast cells were grown in glucose (Glu) or galactose (Gal) under constant white-light (LL) or constant darkness (DD) conditions. (D) Comparison of the traditional Gal/Glu induction ratio in respect to LL/DD induction achieved with FUN-LOV. The average from three biological replicates (*n* = 3) with its standard deviation is shown in panels C and D.

We then explored the capability of the FUN-LOV switch to regulate heterologous protein production upon light stimulation. Thus, the yeast codon-optimized version of the Cannabis sativa limonene synthase (LS) gene was cloned in a pYES2 plasmid, allowing *P*_*GAL1*_ promoter control and V5 tagging of the protein for Western blot analysis (Fig. 4A). This protein is involved in the production of limonene, a compound widely used by the food and household product industries to increase lemon scent in their products (27). Western blot analysis showed that yeast cells expressing the FUN-LOV system achieved an LS induction of 110-fold (LL/DD), which is 2.5 times higher than the average induction (44-fold) achieved, in our hands, by galactose. Notably, FUN-LOV yields lower background levels under DD conditions, particularly compared to the LS levels in the off state of the galactose/glucose systems (Fig. 4B, C, and D). Furthermore, when we evaluated the effect of LL on the temporal expression of proteins by exposing the cells to 2-h pulses of white light or blue light, high levels of protein expression were obtained, with no differences between the two light sources in two different genetic backgrounds (Fig. S5). Overall, FUN-LOV allows high levels of heterologous protein expression upon light stimulation, with low background expression in darkness, and reaching induction levels surpassing chemical induction approaches.

10.1128/mBio.00626-18.5 FIG S5  FUN-LOV-controlled expression of a heterologous protein. (A to C) Expression of V5-tagged limonene synthase (LS) was analyzed by Western blotting (A) and quantified with respect to membrane-stained proteins (B) under three conditions: after 2 h of blue-light pulse (BLP), after 2 h of white-light pulse (WLP), and in constant darkness (DD). The fold induction for LS expression was calculated after the BLP and WLP with respect to DD (C). The experiments were carried out in the BY4741 wild-type strain. (D to F) Similar experiments as those in panels A to C performed in the BY4741 *gal4Δ-gal80Δ* genetic background. Download FIG S5, PDF file, 0.1 MB. Copyright © 2018 Salinas et al. 2018 Salinas et al. This content is distributed under the terms of the Creative Commons Attribution 4.0 International license.

### Modulation of yeast flocculation by light.

**FIG 5:**
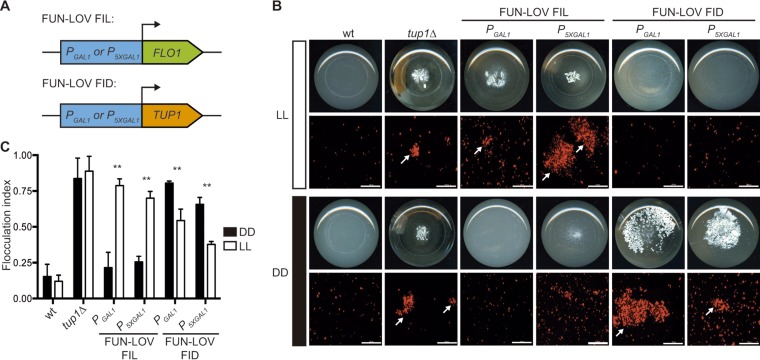
The FUN-LOV switch allows precise control of flocculation by light conditions. (A) FUN-LOV-dependent expression of *FLO1* or *TUP1* genes allows flocculation to occur in light (FIL) or in darkness (FID). (B) FIL and FID phenotypes observed as macroscopic cell aggregation in culture flasks (bottom view) and fluorescence microscopy of cells expressing *mCherry* under the *P*_*TDH3*_ constitutive promoter. Experiments were performed under constant white-light (LL) or constant darkness (DD) conditions. The BY4741 wild-type (wt) and BY4741 *tup1Δ* strains were utilized as negative and positive controls of flocculation, respectively. Cell aggregates are indicated by arrows; bar, 100 µm. (C) Quantification of the FIL and FID phenotypes observed in panel B by calculating the flocculation index. Statistically significant differences between LL and DD conditions are indicated as ** (*t* test, *P* < 0.01).

Finally, we sought to apply the FUN-LOV system to optogenetically modulate a biotechnologically relevant process such as flocculation. We rationalized that direct overexpression of the main genes responsible for flocculation (*FLO1* or *FLO11*) would trigger such a phenotype, in agreement with previous reports for these genes (23, 28). On the other hand, low expression of the yeast transcriptional corepressor *TUP1* should lead to upregulation of its targets (29), among which is *FLO1*, therefore triggering strong flocculation. Initially, we swapped the endogenous promoters of *FLO1*, *FLO11*, and *TUP1* genes for *P*_*GAL1*_ and *P*_*5XGAL1*_ promoters (Fig. 5A). We confirmed the correct replacement of the endogenous promoters and analyzed the behavior of the resulting strains by growing them under conditions of galactose and glucose as carbon sources. Using bright-field microscopy, we observed strong cell aggregation in strains carrying *P_GAL1_-FLO1* in galactose but not in glucose but moderate aggregation in the ones bearing *P_GAL1_-FLO11* (Fig. S6). As expected, strains carrying *P_GAL1_-TUP1* showed strong cell aggregation in glucose but not in galactose, confirming the flocculation phenotype caused by the lack of *TUP1* expression (Fig. S6). Then, we episomally incorporated the FUN-LOV system into the strains carrying the swapped promoters. The strains with *P*_*GAL1*_-*FLO1* and carrying the FUN-LOV system demonstrated high levels of cell aggregation in LL and low or no aggregation in DD (Fig. S7). We designated this FUN-LOV-controlled phenotype flocculation in light (FIL). Compared to the FUN-LOV control of *FLO1*-driven flocculation, only a modest FIL phenotype was observed in LL for strains with *P_GAL1_-FLO11*. These phenotypes were confirmed by macroscopic observation, as well as by fluorescence microscopy of yeast cells constitutively expressing *mCherry*, and by calculating the flocculation index of each strain (Fig. 5B and C and S8). On the other hand, strains with FUN-LOV control of *P_GAL1_-TUP1* revealed high levels of cell aggregation in DD but not in LL (Fig. 5B and C and S7). This phenotype was designated flocculation in darkness (FID).

10.1128/mBio.00626-18.6 FIG S6  Galactose and glucose control of the flocculation phenotype. (A and B) Strains with *FLO1*, *FLO11*, and *TUP1* promoter swapping (*P*_*GAL1*_ and *P*_*5XGAL1*_) were grown in galactose and glucose as carbon sources, and the flocculation phenotype was assayed by bright-field microscopy (A) and macroscopic analyses of the culture flasks (bottom view) (B). BY4741 and BY4741 *flo1Δ* strains were used as negative controls of flocculation, whereas the BY4741 *tup1Δ* strain was used as a positive control of flocculation. Bars, 100 µm at ×20 magnification and 20 µm at ×60 magnification. Download FIG S6, PDF file, 1.1 MB. Copyright © 2018 Salinas et al. 2018 Salinas et al. This content is distributed under the terms of the Creative Commons Attribution 4.0 International license.

10.1128/mBio.00626-18.7 FIG S7  FUN-LOV control of flocculation in light (FIL) and flocculation in darkness (FID) phenotypes. (A and B) Strains with *FLO1*, *FLO11*, and *TUP1* promoter swapping (*P*_*GAL1*_ and *P*_*5XGAL1*_) and carrying the FUN-LOV system were grown under constant light (LL) and constant darkness (DD) conditions, and the flocculation phenotype was assayed by bright-field microscopy (A) and pictures of the culture flasks (bottom view) (B). The BY4741 *wt* and BY4741 *flo1Δ* strains were used as negative controls of flocculation, whereas the BY4741 *tup1Δ* strain was used as a positive control of flocculation. Bars, 100 µm at ×20 magnification and 20 µm at ×60 magnification. Download FIG S7, PDF file, 1.2 MB. Copyright © 2018 Salinas et al. 2018 Salinas et al. This content is distributed under the terms of the Creative Commons Attribution 4.0 International license.

10.1128/mBio.00626-18.8 FIG S8  Quantification of light-regulated flocculation by FUN-LOV. (A) Flocculation in light (FIL) and flocculation in darkness (FID) were monitored as macroscopic phenotypes by observing cell aggregation in culture flasks (bottom view), as well as by fluorescence microscopy of cells expressing *mCherry* under a constitutive promoter (*P*_*TDH3*_). Experiments were performed under constant white light (LL) and constant darkness (DD). wt yeast cells (BY4741) as well as *flo1Δ* and *flo11Δ* strains were utilized as negative controls, whereas the *tup1Δ* strain was used as a positive control of flocculation. Cellular aggregations are highlighted with arrows. Bar, 100 µm. (B) Quantification of the flocculation index of the FIL and FID phenotypes depicted in panel A, where the symbol ** represents a significant statistical difference between LL and DD conditions (*t* test; **, *P* < 0.01). Download FIG S8, PDF file, 0.5 MB. Copyright © 2018 Salinas et al. 2018 Salinas et al. This content is distributed under the terms of the Creative Commons Attribution 4.0 International license.

**FIG 6:**
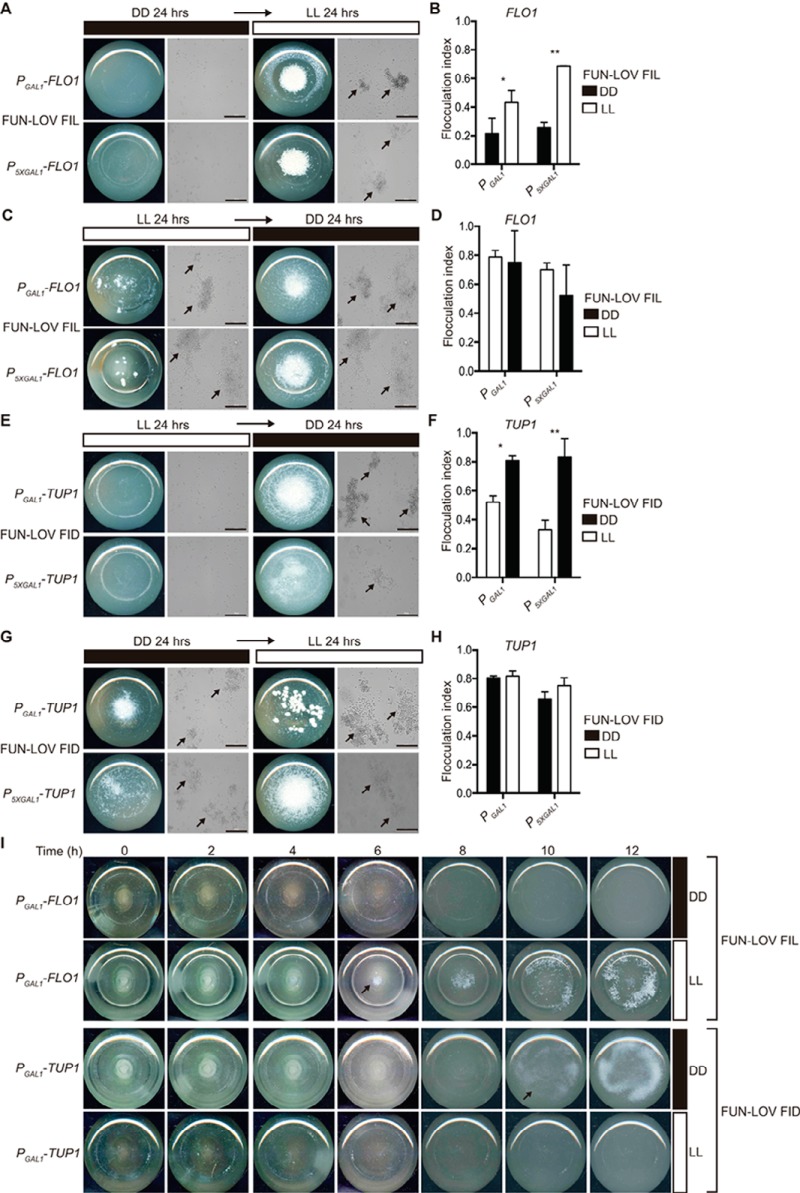
Reversibility and temporality of the different flocculation phenotypes. (A to D) The reversion of the FIL phenotype for strains with *FLO1* promoter swapping and carrying the FUN-LOV system was assayed after 24 h of incubation in constant darkness (DD) and then transfer for 24 h to constant light (LL) (A and B); the opposite regimen was also assayed, with 24 h in LL and 24 h in DD (C and D). Cellular aggregation was recorded with pictures of the bottom view of the culture flasks and bright-field microscopy (A and C). Yeast aggregates are highlighted with arrows; bar, 100 µm. The flocculation index was assayed for the same set of strains and culture conditions (B and D). Single and double asterisks represent statistically significant differences between LL and DD conditions (*t* test; *, *P* < 0.05, and **, *P* < 0.01). (E to H) Reversion of the FID phenotype for strains with *TUP1* promoter swapping and carrying the FUN-LOV system was assayed under the same experimental conditions as in panels A to D, starting from the nonflocculating condition (LL) to the flocculating condition (DD) (E); the opposite regimen was also assayed (G). The flocculation index was assayed for the same set of strains and culture conditions (F and H). (I) Time course of the FIL and FID phenotypes for the strains with *FLO1* and *TUP1* promoter swapping (by *P*_*GAL1*_) and carrying the FUN-LOV system. The cellular aggregation was recorded with pictures of the bottom view of the culture flasks every 2 h under DD and LL conditions. The arrows highlight the time point where the macroscopic flocculation phenotype started to be visible.

The reversibility of the flocculation phenotype was evaluated under different conditions of LL and DD. Growth of *FLO1* FIL strains during 24 h in DD followed by 24 h in LL resulted in strong flocculation (Fig. 6A and B), whereas the opposite treatment—24 h in LL followed by 24 h in DD—revealed strong flocculation in LL without reversion of the phenotype after the transfer to DD (Fig. 6C and D). In the case of *TUP1* FID strains, there was no flocculation in LL during the initial 24 h of incubation, followed by strong flocculation after the transfer to DD (Fig. 6E and F). As expected, no reversion was observed for the phenotype when cells were incubated for 24 h in DD and then transferred to LL (Fig. 6G and H). Finally, we evaluated the time necessary to induce the FIL and FID phenotypes in yeast cultures. The results showed that the FIL phenotype becomes readily visible after 6 h of light stimulation, whereas the FID phenotype can be observed after 10 h of incubation in the dark (Fig. 6I). Overall, depending on the genetic wiring of FUN-LOV, yeast flocculation can be triggered by light (FIL phenotype) or by its absence (FID phenotype). These phenotypes showed no reversibility, and the time of appearance exhibited different temporalities.

## DISCUSSION

Based on the LOV domains of two blue-light photoreceptors from the fungus N. crassa (proteins WC-1 and VVD), we developed a new optogenetic switch named FUN-LOV. We set up FUN-LOV in yeast cells, since this microorganism is a model eukaryotic system for genetics and molecular biology studies and a powerful platform for biotechnology, including applications such as production of high-value metabolites and proteins (30, 31). Using yeast as a biological chassis, FUN-LOV showed three singular features: (i) high levels of gene expression with a broad dynamic range and temporal resolution (Fig. 1, 2 and 3); (ii) low background expression under dark conditions (Fig. 1C); and (iii) the control of biotechnologically relevant phenotypes in yeast, such as heterologous protein expression (Fig. 4) and flocculation (Fig. 5 and 6). Those characteristics achieved by FUN-LOV increase its potential uses under industrial or high-volume bioprocess conditions.

Light has been positioned in previous years as a promising tool to control gene expression due to its reduced toxic effects on cells, low costs compared to chemical inducers, and the ability to confer spatiotemporal modulation of biological processes (3). In this work, we selected yeast as a biological platform for the implementation of FUN-LOV, ensuring the orthogonal effect of light over gene expression. Importantly, it has been described that extremely high intensities of blue, green, and white lights can affect yeast growth, producing impaired respiration, increased levels of reactive oxygen species, and upregulation of the stress response (32). In this sense, FUN-LOV reached the maximum level of gene expression at 20 µmol m^−2^ s^−1^ of blue-light intensity, consistent with previous reports where between 2 and 24 µmol m^−2^ s^−1^ of blue light is enough for VVD photocycle activation (33). Utilizing rather low light levels could be considered an advantage of our system, as it reduces the possibility of undesirable effects of high light on yeast physiology and metabolism. Nevertheless, it is interesting that the dynamics of the luciferase reporter under blue light show a drop not seen in white light (i.e., Fig. 1D and E), a feature that is not observed in the luciferase data prior to normalization by optical density (OD) (see Fig. S2 in the supplemental material).

The first optogenetic switch developed and implemented in yeast was based on the red-light-dependent interactions of the photoreceptor phytochrome B (PhyB) and its interacting protein PIF3 from Arabidopsis thaliana (6). Ever since, this switch has been adapted in yeast for different purposes such as reconstitution of protein activities (7), subcellular protein localization and control of cell polarity and budding phenotypes (34, 35), and reporter gene expression (yellow fluorescent protein [YFP] or green fluorescent protein [GFP]) (19, 36). Recently, a new red-light optogenetic switch also based on PhyB-PIF3 interaction (named PhiReX), which overcomes the obstacle of external addition of the chromophore phycocyanobilin (PCB), has been implemented and tested in yeast (36). These types of toggle switches can be quite powerful as they provide activation with a given wavelength (red) to then be turned off by another one (far-red). Yet, in some cases that contrasts with the simplicity of blue-light switches, which can be readily activated by blue or white light, while they can be turned off with fast kinetics upon transfer to the dark, without the need of extra specific wavelengths.

In general, multiple blue-light optogenetic switches have been implemented in several biological platforms, utilizing a wide repertoire of biological parts and resulting in diverse expression levels. The latter has been monitored by different reporter genes, a fact that complicates the direct comparison between systems (22). In yeast, one of the most widely used optogenetic switches is based on the blue-light-dependent interaction of the photoreceptor cryptochrome 2 (CRY2) and its interacting protein CIB1 from A. thaliana, which has allowed light-controlled reporter gene expression (20), heterologous expression of YFP or mCherry (37, 38), and cell cycle modulation (19). Besides cryptochromes, blue-light photoreceptors containing LOV domains have been also used in yeast optogenetics. The LOV2 domain from Avena sativa phototropin 1 (AsLOV2) was utilized for light-controlled caging of peptides and β-galactosidase expression in yeast (39). Similarly, the LOV2 domain from A. thaliana phototropin 1 (AtLOV2) has been also used for blue-light control of the cell cycle progression and expression of conditional essential genes (40, 41).

Fungal blue-light photoreceptors containing LOV domains have been seldom utilized in optogenetics, and their uses have not been exploited in yeast, regardless of the functional conservation between fungal and plant LOV domains (42). Only the protein VIVID (VVD) from the fungus N. crassa has been used in optogenetics switches, permitting light-controlled expression of transgenes in mice and mammalian cells (13, 14). The latter optogenetic switch, named GAVPO and commonly known as the “LightOn” system, is based on the homodimerization of VVD LOV domains upon light stimulation and also includes components such as a GAL4 DNA binding domain (GAL4-DBD) and a p65 transactivation domain, reaching 200- to 300-fold induction, as measured by a luciferase reporter in mammalian cells (14). Thus, the fact that “LightOn” shares several components with FUN-LOV (VVD and GAL4-DBD) is a positive feature that supports future adaptability of FUN-LOV beyond yeast, including mammalian systems.

Optogenetic systems based on the homodimerization of LOV domains have shown proper functionality for light-controlled gene expression. However, they have limited applications in light-activated control of subcellular protein localization, due to the possibility of homodimerization of one of the target proteins. This problem has been nicely solved using the “Magnets” system, where the VVD LOV domain has been engineered to recognize a VVD partner with the opposite electrostatic charge, showing successful subcellular protein localization (43) when tested in mammalian cell lines (44). In this sense, it is worth mentioning that VVD/VVD or WC-1/WC-1 self-dimerization could be decreasing the induction levels achieved by FUN-LOV, as it would reduce the proportion of WC-1/VVD pairing, which is the one needed to reconstruct a chimeric transcription factor that allows expression of a target gene. Therefore, future efforts will seek to implement the “Magnets” strategy in our system to avoid the formation of nonfunctional homodimers, boosting induction to even higher levels.

Indeed, in N. crassa not only has the protein VVD the capacity to self-dimerize upon light stimulation, but additionally VVD interacts with the protein WC-1, a LOV-LOV interaction that allows photoadaptation to different light intensities (17, 45). Importantly, WC-1 is a GATA transcription factor and also a blue-light photoreceptor containing a LOV domain, participating (with WC-2 protein) in the White-Collar complex (WCC), which acts as a positive element in the circadian clock of N. crassa (46). Therefore, when it comes to LOV, finding the right partner may improve the performance of the system, as evidenced by the VVD/WC-1 combination. This naturally occurring LOV-LOV interaction of WC-1 and VVD opens the door for the development of novel optogenetic switches based on different LOV pairs, such as WC-1/WC-1 from either *Neurospora* or other fungi; considering the diversity of such sequences in different fungal genomes, such LOV domains are a promising source for novel optogenetic switches such as the one presented here. Moreover, the ability to easily assess functional interactions between full-length (as utilized in FUN-LOV) or N-terminally truncated VVD (as in GAPVO) and homo- or hetero-partners in yeast can help advance structure-function studies allowing evaluation of the effect of different mutations in LOV-LOV interactions.

In conclusion, in this work we reported the implementation in yeast of a novel optogenetic switch, FUN-LOV, which provides accurate and strong light-controlled gene expression, exemplified also by the regulation of two phenotypes of biotechnological relevance, such as heterologous protein expression and flocculation. The current challenge is to successfully scale up this technology for industrial bioprocesses, under conditions where high culture densities could preclude efficient light delivery to all yeast cells. Importantly, in addition to its applicability to producing high-value metabolites or heterologous proteins, its low background and broad dynamic range make FUN-LOV a powerful tool to exquisitely regulate the expression of any gene of interest and to probe complex biological phenomena. In addition, its modular design can help the implementation of different optologic gates, by combining it with optogenetic switches for other light wavelengths (22), in order to rewire, build, or perturb complex gene regulatory networks in yeast. Notably, as FUN-LOV utilizes a Gal4-DBD, it could be readily domesticated in other systems such as mammals, or *Drosophila*, where Gal4 orthogonal control of gene expression has proven extremely successful.

## MATERIALS AND METHODS

### Yeast strains, medium, and culture conditions.

Saccharomyces cerevisiae strains BY4741 wild type (wt) (*MATa his3Δ1 leu2Δ0 met15Δ0 ura3Δ0*) and BY4741 with *GAL4* and *GAL80* deletions (*MATa his3Δ1 leu2Δ0 met15Δ0 ura3Δ0 gal4Δ*::*NatMx gal80Δ*::*HphMx*) were used as genetic background for gene deletions and promoter swapping. The strains used and generated in this work were maintained in YDPA medium (2% glucose, 2% peptone, 1% yeast extract, and 2% agar) at 30°C, and their genotypes are listed in Table 1. Strains carrying plasmids with auxotrophic markers were maintained in synthetic complete (SC) medium (0.67% yeast nitrogen base without amino acids, 2% glucose, 0.2% dropout mix, and 2% agar) minus the corresponding amino acid (dropout mix). Growth of yeast cultures under different white-light (LL) and darkness (DD) conditions was conducted at 30°C, using 100 µmol m^−2^ s^−1^ of white-light intensity, which was the light intensity provided by the Percival incubators (Percival Scientific, USA). In general, cells were grown in 50 ml of SC medium at 30°C with 130 rpm of shaking in flasks or in 200 µl of SC medium using 96-well plates at 30°C.

**TABLE 1:** Strains of Saccharomyces cerevisiae used and generated in this work

Strain	Genotype	Source
BY4741	*MATa his3Δ1 leu2Δ0 met15Δ0 ura3Δ0*	Euroscarf
*gal4*Δ-*gal80*Δ	BY4741; *gal4*Δ::*NatMx gal80*Δ::*HphMx*	This work
*flo1*Δ	BY4741; *flo1*Δ::*URA3*	This work
*flo11*Δ	BY4741; *flo11*Δ::*URA3*	This work
*tup1*Δ	BY4741; *tup1*Δ::*KanMx*	Euroscarf
*P_GAL1_-FLO1*	BY4741; *P*_*FLO1*Δ_::*KanMxRV-P*_*GAL1*_	This work
*P_5XGAL1_-FLO1*	BY4741; *P*_*FLO1*Δ_::*KanMxRV-P*_*5XGAL1*_	This work
*P_GAL1_-FLO11*	BY4741; *P*_*FLO11*Δ_::*KanMxRV-P*_*GAL1*_	This work
*P_5XGAL1_-FLO11*	BY4741; *P*_*FLO11*Δ_::*KanMxRV*-*P*_*GAL1*_	This work
*P_GAL1_-TUP1*	BY4741; *P*_*TUP1*Δ_::*KanMxRV-P*_*GAL1*_	This work
*P_5XGAL1_-TUP1*	BY4741; *P*_*TUP1*Δ_::*KanMxRV-P*_5X*GAL1*_	This work
*gal3*Δ::*P_GAL1_-Luc*	BY4741; *gal3*Δ::*KanMxRV-P_GAL1_-Luc*	This work
*gal3*Δ::*P_5XGAL1_-Luc*	BY4741; *gal3*Δ::*KanMxRV-P_5XGAL1_-Luc*	This work
*gal4*Δ-*gal80*Δ *P_GAL1_-FLO1*	BY4741; *gal4*Δ-*gal80*Δ *P*_*FLO1*Δ_::*KanMxRV-P*_*GAL1*_	This work
*gal4*Δ-*gal80*Δ *P_GAL1_-FLO11*	BY4741; *gal4*Δ-*gal80*Δ *P*_*FLO11*Δ_::*KanMxRV-P*_*GAL1*_	This work
*gal4*Δ-*gal80*Δ *P_GAL1_-TUP1*	BY4741; *gal4*Δ-*gal80*Δ *P*_*TUP1*Δ_::*KanMxRV-P*_*GAL1*_	This work
*gal4*Δ-*gal80*Δ *gal3*Δ::*P_GAL1_-Luc*	BY4741; *gal4*Δ-*gal80*Δ *gal3*Δ::*KanMxRV-P_GAL1_-Luc*	This work
*gal4*Δ-*gal80*Δ *gal3*Δ::*P_5XGAL1_-Luc*	BY4741; *gal4*Δ-*gal80*Δ *gal3*Δ::*KanMxRV-P_5XGAL1_-Luc*	This work

### Illumination conditions.

Blue-light (BL) experiments under microcultivation conditions were carried out using an LED lamp (growth LED, model i5038, 2 W of potency, and 38 LED pieces) at 20 µmol m^−2^ s^−1^ of light intensity, except in the data presented in Fig. 3C, where 40 µmol m^−2^ s^−1^ was utilized. In the case of white light (LL), experiments were performed with an LED lamp (General Electric; type A60, 10 W of potency) at 100 µmol m^−2^ s^−1^ of light intensity. Light intensity was measured using a quantum light meter, model LightScout (Spectrum Technologies Inc., USA), which measures the photosynthetically active radiation (PAR) light between 400 and 700 nm. Additionally, light intensity was regulated by modifying the distance between the 96-well plate and the light source. The spectrum of the white and blue LED lamps was determined using an HR2000 high-resolution spectrometer (Ocean Optics, USA), showing that the two light sources have similar peaks at 460 nm. The heterologous protein expression and flocculation experiments were conducted in a Percival incubator, which delivered 100 µmol m^−2^ s^−1^ of light intensity. Manipulation of strains in the “dark” was conducted in a light-tight room equipped with red safety lights.

### Plasmid construct and strain generation.

Plasmids pBM1 and pBM2 carrying the LOV domains of WC-1 (WC-1 LOV-BD) and VVD (VVD-AD, which actually contains the full-length VVD and not just VVD-36) were generously provided by the Brunner lab (17). The plasmids used and generated in this work are shown in Table 2. The FUN-LOV components were cloned into pRS423 (WC-1 LOV plus GAL4-DBD) and pRS425 (VVD plus GAL4-AD) plasmids for *HIS3* and *LEU2* auxotrophic selection, respectively. All the cloning experiments were performed and genetic constructs were generated using yeast recombinational cloning *in vivo* assembly (47, 48).

**TABLE 2:** Plasmids assembled in this work using yeast recombinational cloning

Plasmid	Construct
pRS423-WC-1	*P_ADH1_-WC-1 LOV-GAL4 DBD-ADH2_ter_*
pRS425-VVD	*P_ADH1_-VVD-GAL4 AD-ADH2_ter_*
pRS426-*P_GAL1_-Luc*	*KanMxRV-P_GAL1_-Luc-CYC1_ter_*
pRS426-*P_5XGAL1_-Luc*	*KanMxRV-P_5XGAL1_-Luc-CYC1_ter_*
pRS426-*P*_*GAL1*_	*KanMxRV-P_GAL1_*
pRS426-*P*_*5XGAL1*_	*KanMxRV-P_5XGAL1_*
pRS426-*mCherry*	*P_TDH3_-mCherry-CYC1_ter_-HphMx*
pRS426-*P*_*ADH1−*_*Luc*	*P_ADH1_-Luc-CYC1_ter_*
pRS426-*P_TEF1_-Luc*	*P_TEF1_-Luc-CYC1_ter_*
pRS426-*P_TDH3_-Luc*	*P_TDH3_-Luc-CYC1_ter_*

The synthetic version of the *P*_*GAL1*_ promoter, called *P*_*5XGAL1*_, carrying five DNA binding sites for the Gal4 transcription factor (GAL4-UAS), was synthesized using the Genewiz gene synthesis service. In the construct assembly, the *P*_*GAL1*_ and *P*_*5XGAL1*_ promoters were amplified by PCR using Phusion Flash high-fidelity PCR master mix (Thermo Scientific, USA). Additionally, the kanamycin (*KanMx*) antibiotic resistance cassette was added in the reverse direction upstream of each promoter (*KanMxRv-P*_*GAL1*_ or *KanMxRv-P*_*5XGAL1*_) using yeast recombinational cloning (48). The complete genetic construct (*KanMxRv-P*_*GAL1*_ or *KanMxRv-P*_*5XGAL1*_) was used to transform the BY4741 wt and BY4741 *gal4Δ-gal80Δ* strains. The complete genetic constructs (*KanMxRv-P*_*GAL1*_ or *KanMxRv-P*_*5XGAL1*_) were amplified by PCR using a Phusion Flash high-fidelity PCR master mix (Thermo Scientific, USA) and 70-bp primers for direct homologous recombination on the target locus, allowing the swapping of the endogenous promoter region. The promoter swapping of *FLO1*, *FLO11*, and *TUP1* was confirmed by PCR under standard conditions; primers used for plasmid assembly, promoter swapping, and promoter swapping confirmations are shown in Table S1 in the supplemental material. The same assembly procedure was followed to construct different versions of the luciferase reporter gene under the control of *P*_*GAL1*_ and *P*_*5XGAL1*_ promoters. The deletion of *GAL4* or *GAL80* or the integration of the luciferase reporter at the *GAL3* locus was carried out using one-step PCR deletion by recombination (49). Primers used for gene deletion and reporter gene integration in the genome and its confirmation are listed in Table S1.

10.1128/mBio.00626-18.9 TABLE S1  Primers used in this work. Download TABLE S1, PDF file, 0.1 MB. Copyright © 2018 Salinas et al. 2018 Salinas et al. This content is distributed under the terms of the Creative Commons Attribution 4.0 International license.

### Luciferase *in vivo* expression and Western blot assay.

We used a previously described destabilized version of firefly luciferase for real-time monitoring of gene expression in yeast (50). We cloned into plasmid pRS426 the luciferase reporter gene under the control of the *P*_*GAL1*_ and *P*_*5XGAL1*_ promoters using yeast recombinational cloning as we described above. Real-time luciferase expression was measured under DD, LL, and BL conditions using a Cytation 3 microplate reader (BioTek, USA), which allows the measurement of both optical density at 600 nm (OD_600_) and luminescence of the cell cultures over time. Briefly, the yeast strains were grown overnight in a 96-well plate with 200 µl of SC medium at 30°C under the DD condition, and 10 µl of these cultures was used to inoculate a new 96-well plate containing 290 µl (30-fold dilution) of fresh medium plus 1 mM luciferin. The OD_600_ and the luminescence were acquired every 30 min using a Cytation 3 microplate reader, running a discontinuous kinetic protocol with 30 s of shaking (285 cycles/min) before each measurement. This protocol also allowed illumination of the 96-well plate between each measurement, keeping the plate outside the equipment and exposing it to the light source. Luciferase expression was normalized by OD_600_ of the yeast cultures, and all experiments were performed in six biological replicates (51). Normalization of real-time luciferase levels was necessary as yeast biomass rapidly changed over the length of the experiment, something that may not be necessary when monitoring luciferase from filamentous fungi (52).

Limonene synthase (LS) enzyme cDNA sequence from Cannabis sativa was codon optimized for S. cerevisiae and cloned into pYES2 expression vector. The strain BY4741 wt was cotransformed with pYES2-LS plasmid and the components of the FUN-LOV system. Strains were grown under DD and LL conditions until the OD_600_ was 1, and protein extractions of the yeast cellular pellets were carried out under standard conditions (53). We used 25 µg of total protein from each sample for Western blot assays; detection of the LS was performed using anti-α-V5 primary antibody (Invitrogen, USA) and goat anti-mouse IgG(H+L)-horseradish peroxidase (HRP)-conjugated antibody (Bio-Rad, USA) as secondary antibody (52, 54). All the Western blot experiments were performed in three biological replicates (*n* = 3).

### Gene expression by qPCR.

The gene expression levels for the FUN-LOV component (GAL4-DBD) was measured by real-time PCR (qPCR) in the BY4741 genetic background under the LL condition. Briefly, the yeast strains were grown for 8 h under the LL condition at 30°C in SC medium for RNA extractions. RNA extractions were carried out using Trizol reagent (Thermo Fisher Scientific, USA); the quality and integrity of the total RNA were confirmed by 1% agarose gel and NanoDrop (Thermo Fisher Scientific, USA) quantification. We used 100 ng of total RNA for reverse transcription using Superscript III transcriptase (Invitrogen, USA) under standard conditions. The cDNA was amplified using 2× SensiMix SYBR Hi-ROX kit (Bioline, USA) and using StepOnePlus real-time PCR equipment (Thermo Fisher Scientific, USA). The relative gene expression was calculated using the threshold cycle (2^−ΔΔ*CT*^) method and utilizing two different reference genes (*HEM2* and *TAF10*) (55, 56). All the qPCR experiments were performed in three biological replicates (*n* = 3), and primers used for qPCR amplifications are listed in Table S1.

### Luciferase enzymatic activity.

The luciferase activity assays were carried out in three biological replicates (*n* = 3). The luciferase protein activity was assayed using the luciferase assay system kit (catalog no. E1500; Promega, USA) with modifications. Briefly, the yeast strains were grown for 8 h at 30°C in SC medium and harvested by centrifugation for 5 min at 4,000 × *g*. The cell pellet was disrupted using 200 µl of 2× lysis buffer reagent (Promega, USA) and 200 µl of glass beads in a TissueLyser II equipment (Qiagen, USA) for 3 min. Cells were centrifuged for 5 min at maximum speed, and the supernatant containing the protein extract was recovered and quantified by the Bradford standard method. The luciferase activity was assayed combining 5 µl of the total extracted proteins plus 100 µl of luciferase assay reagent (Promega, USA). The luminescence was immediately recorded in a Cytation 3 microplate reader (BioTek, USA), and it was normalized using the total protein concentration of each sample.

### Flocculation phenotypes.

Strains with promoter swapping in *FLO1*, *FLO11*, and *TUP1* and carrying the FUN-LOV system were evaluated under DD and LL conditions as we previously described. Scans of the flocculation phenotype were taken after 24 h of growth in culture flasks under DD or LL conditions. Additionally, the strains were transformed with a pRS426 plasmid carrying *mCherry* controlled by the *P*_*TDH3*_ promoter. Pictures of yeast cells were taken under bright-field and fluorescence microscopy using a Cytation 3 in microscope mode (BioTek, USA). In the time course experiments, scanning of the culture flask was performed every 2 h.

The flocculation of each strain was quantified by calculating the flocculation index of each strain at OD_600_ (57, 58). The flocculation index was calculated as the OD difference in a yeast culture after 30 min of static incubation: 1 − (final_OD_/initial_OD_). All the flocculation experiments were conducted in three biological replicates (*n* = 3).

### Statistical analysis.

All the statistical analyses were carried out using GraphPad (Prism) software version 6.0.
