# Supplementary figures and images for: Fungal Light-Oxygen-Voltage Domains for Optogenetic Control of Gene Expression and Flocculation in Yeast
Source: mBio. 2018 Jul 31;9(4):e00626-18. doi: 10.1128/mBio.00626-18 (PMC6069114; doi:10.1128/mBio.00626-18)

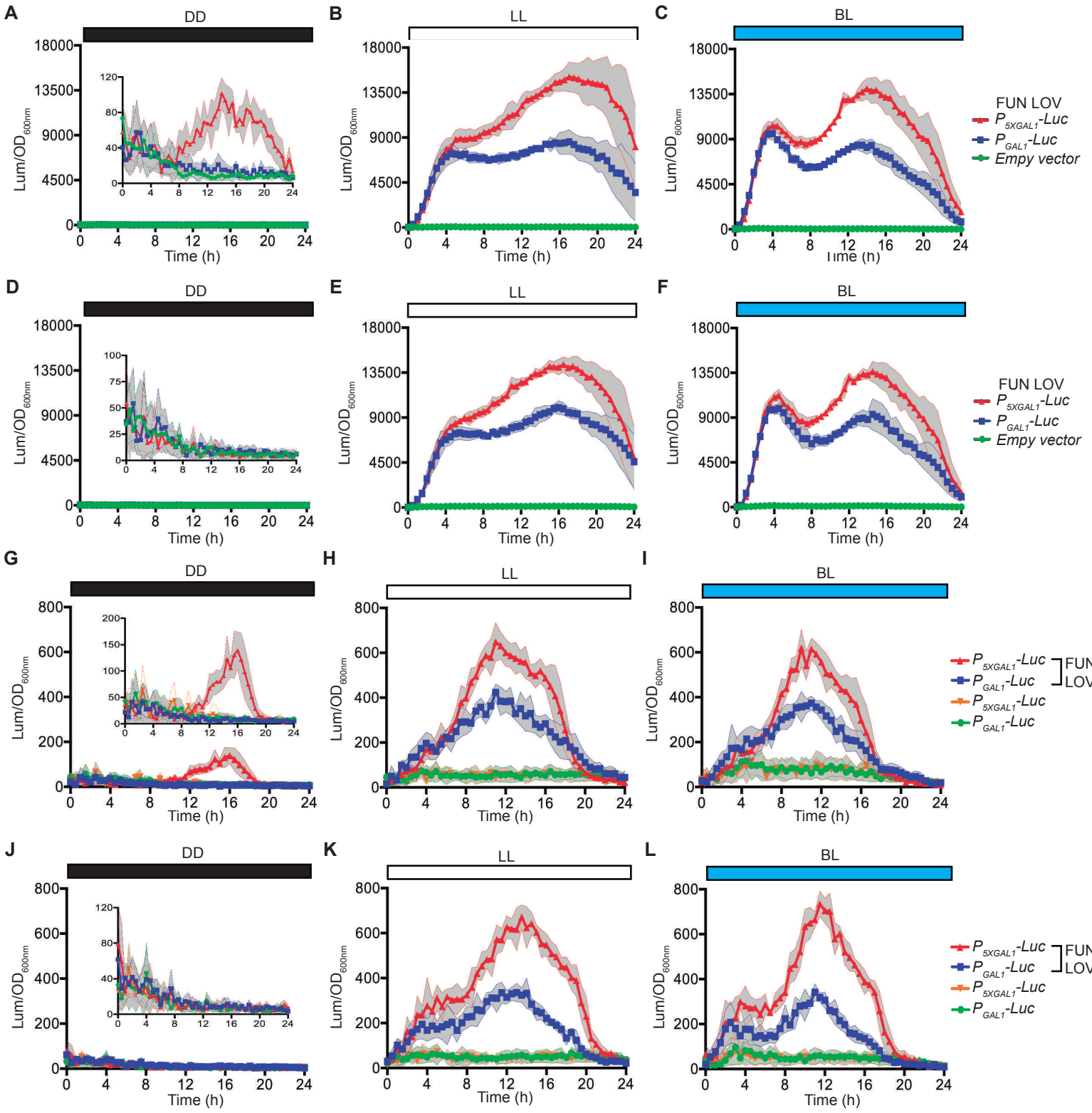

Supplement: FIG S1 [file mbo004183986sf1.pdf]

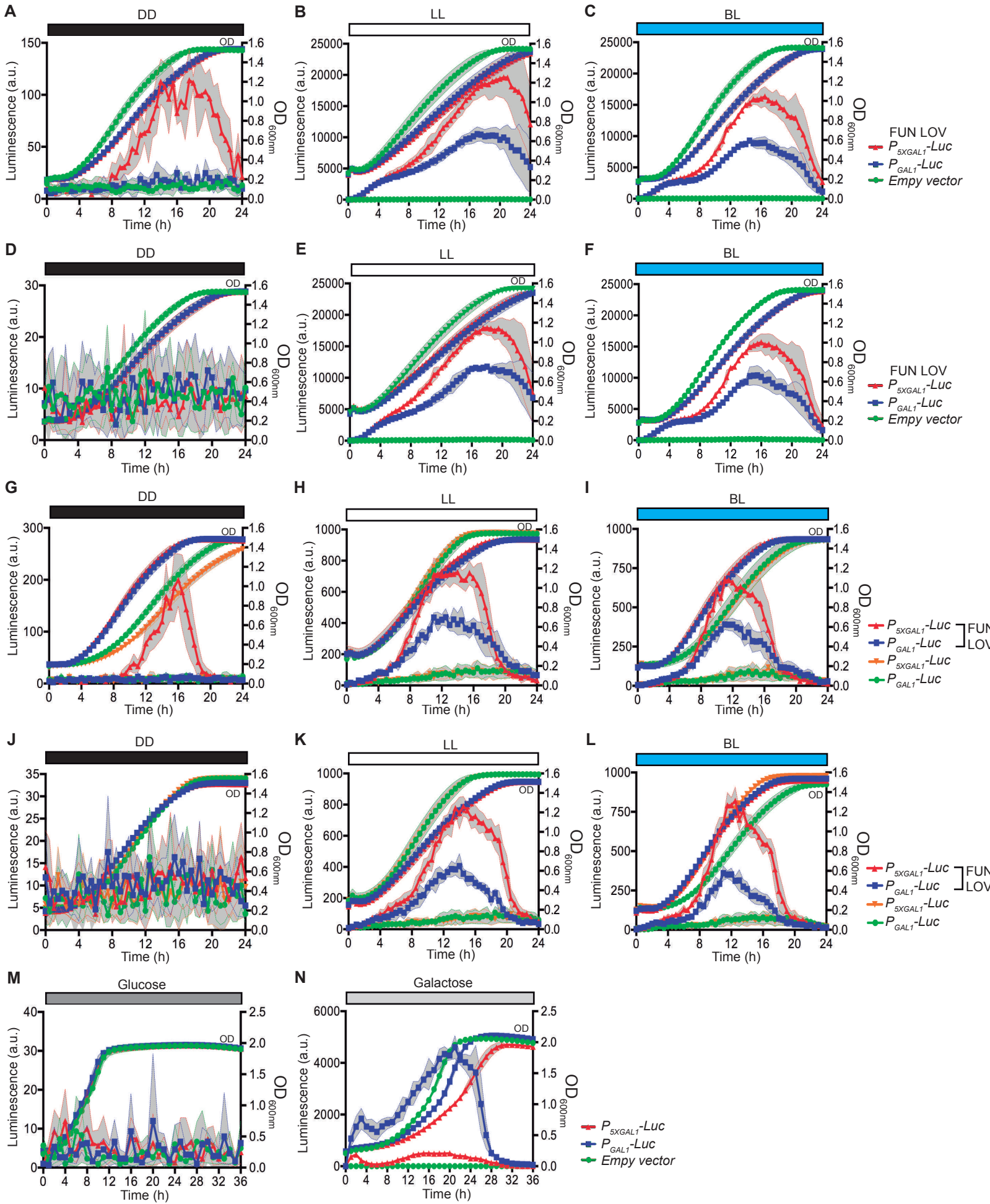

Supplement: FIG S2 [file mbo004183986sf2.pdf]

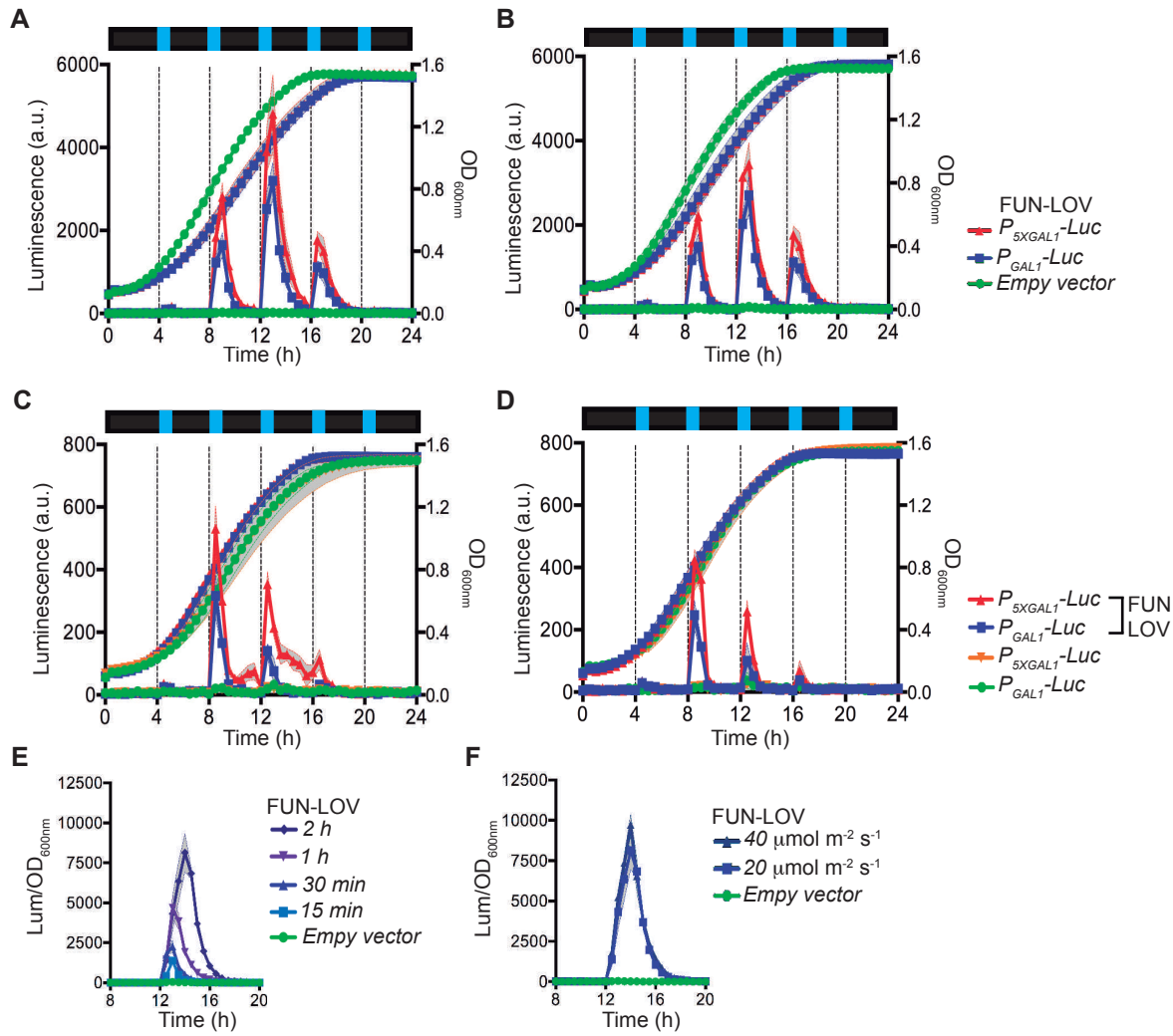

Supplement: FIG S3 [file mbo004183986sf3.pdf]

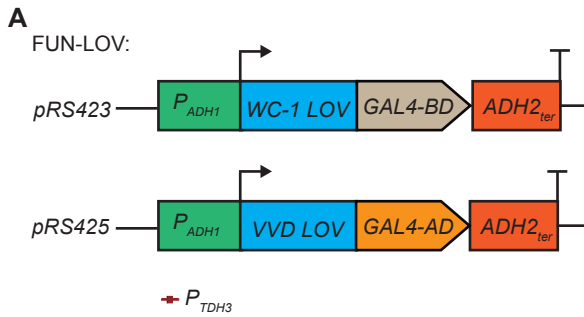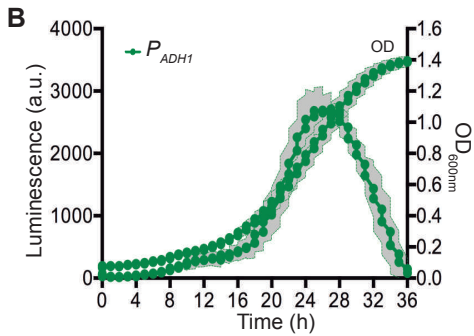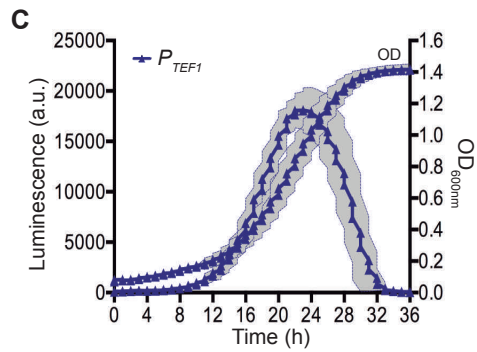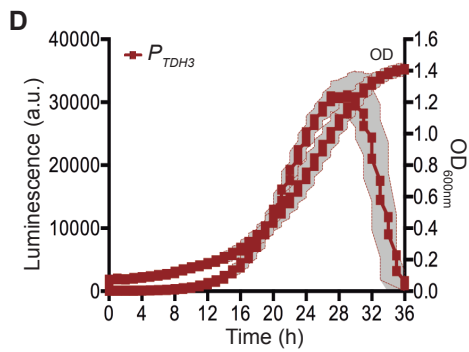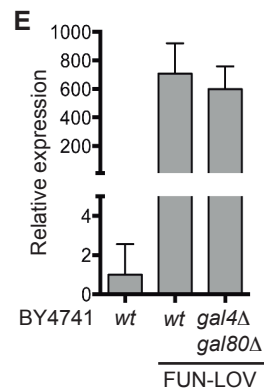

Supplement: FIG S4 [file mbo004183986sf4.pdf]

**A**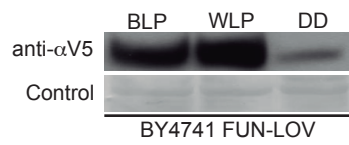**B**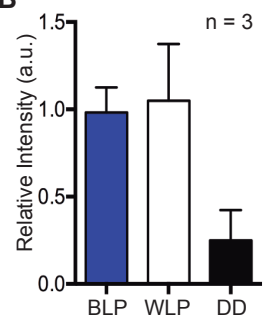**C**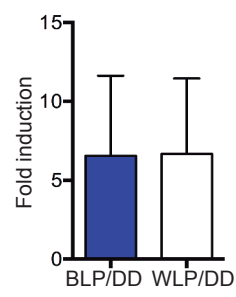**D**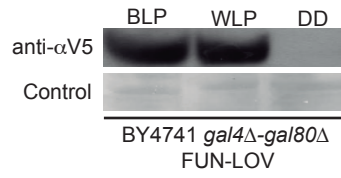**E**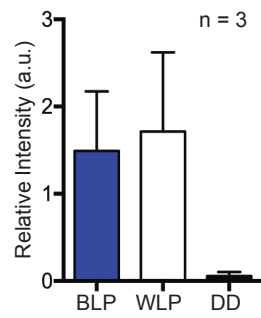**F**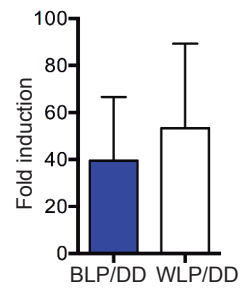

Supplement: FIG S5 [file mbo004183986sf5.pdf]

**A**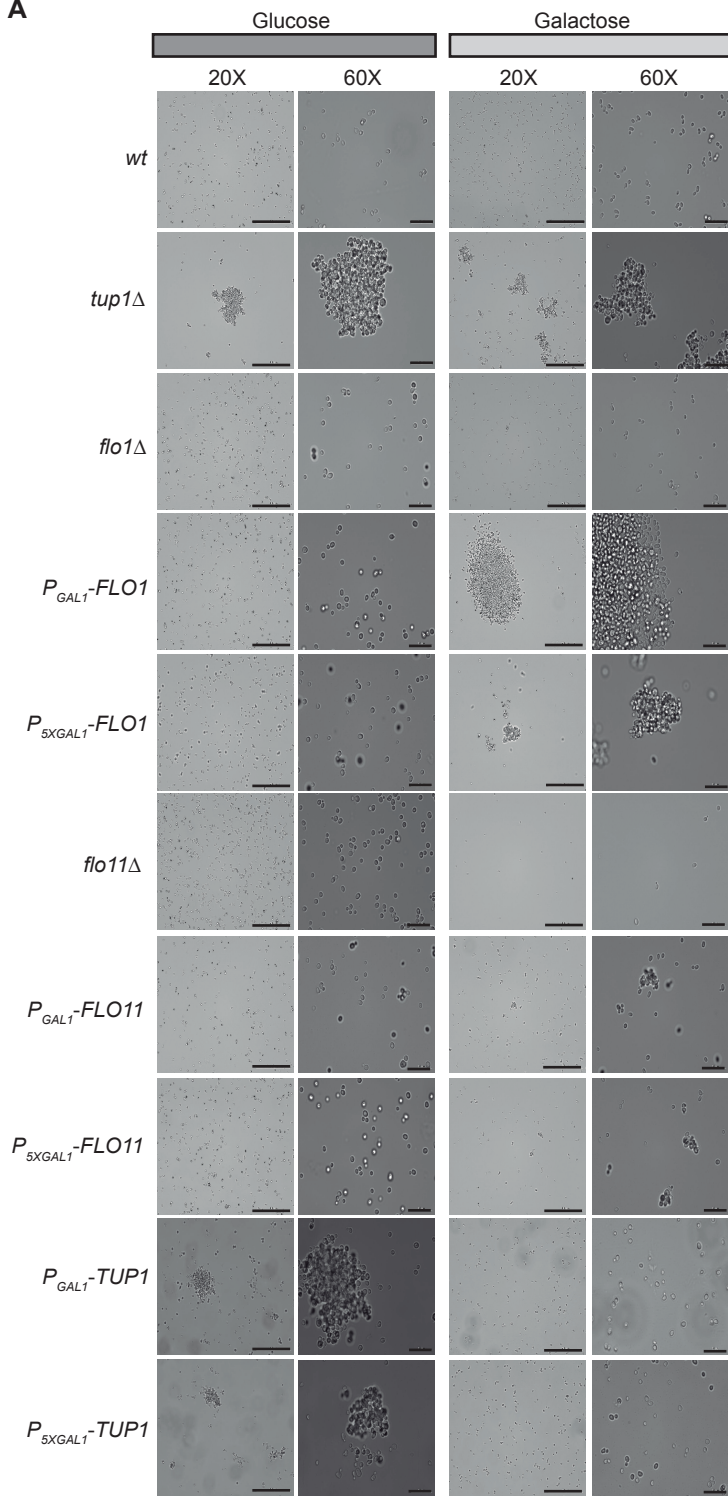**B**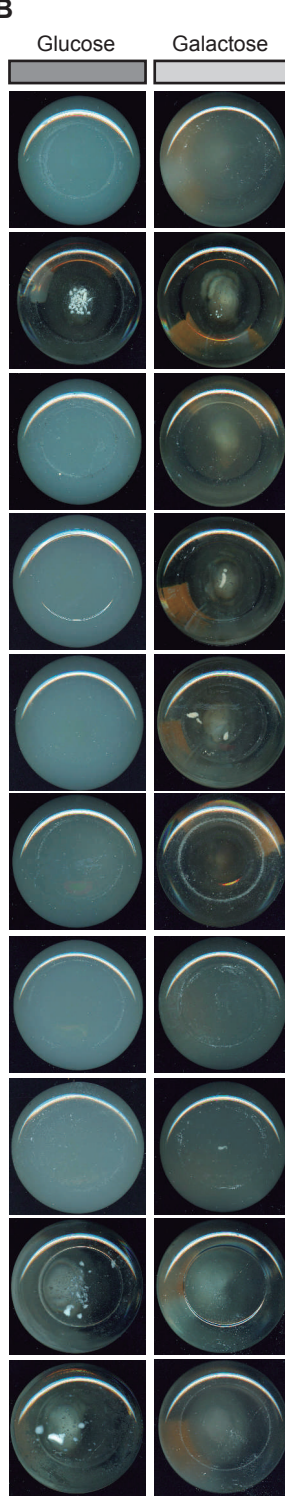

Supplement: FIG S6 [file mbo004183986sf6.pdf]

**A**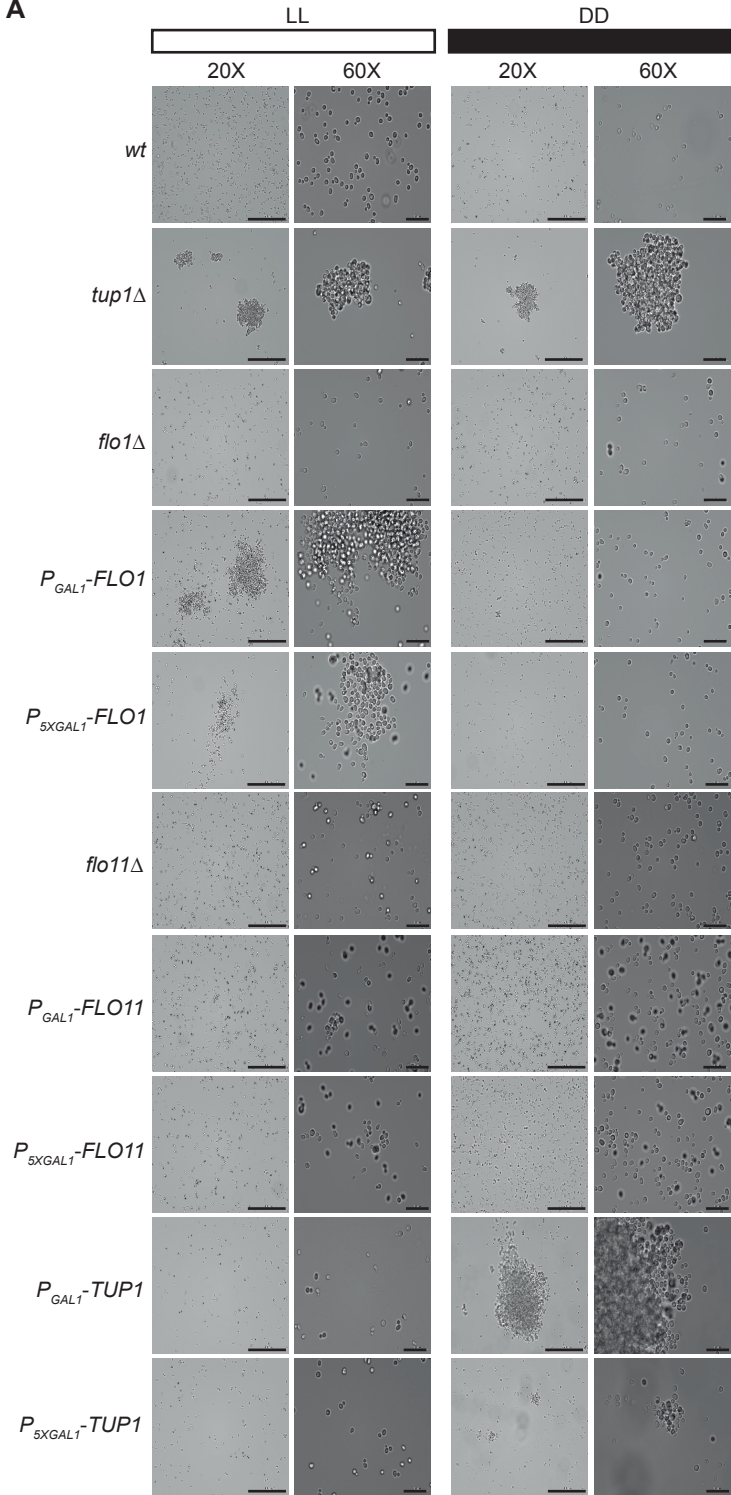**B**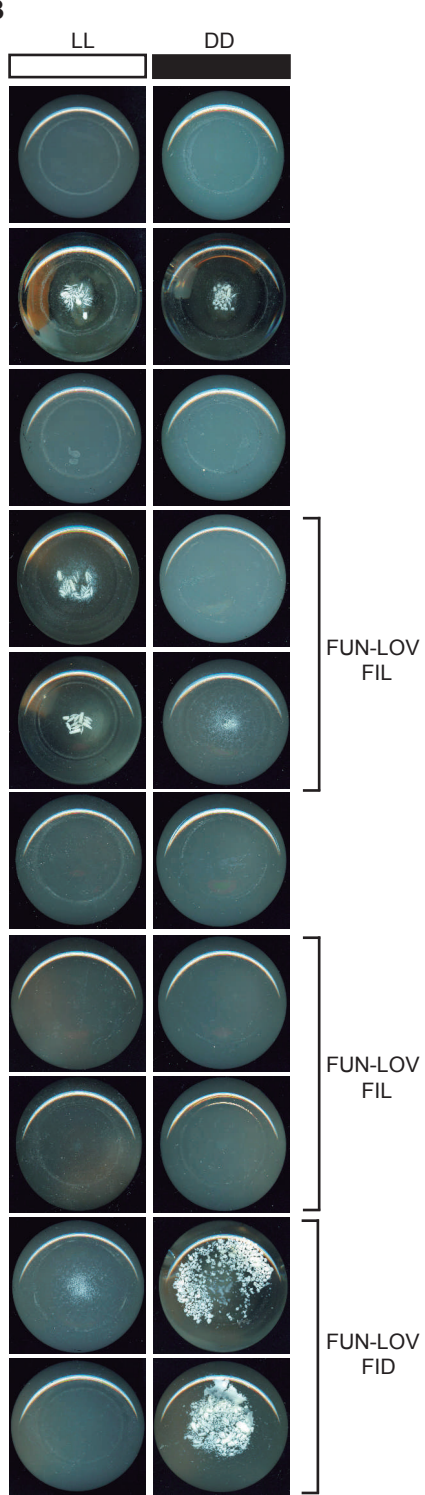

Supplement: FIG S7 [file mbo004183986sf7.pdf]

**A**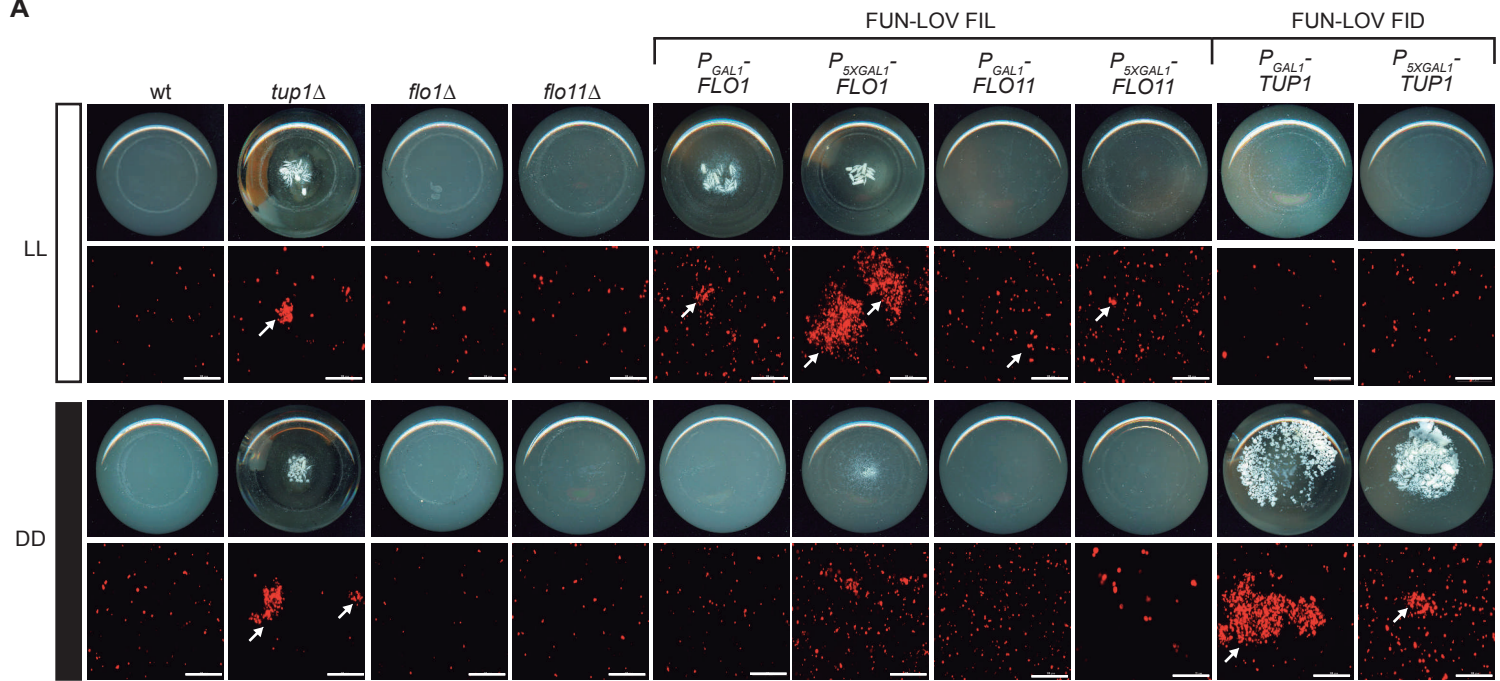**B**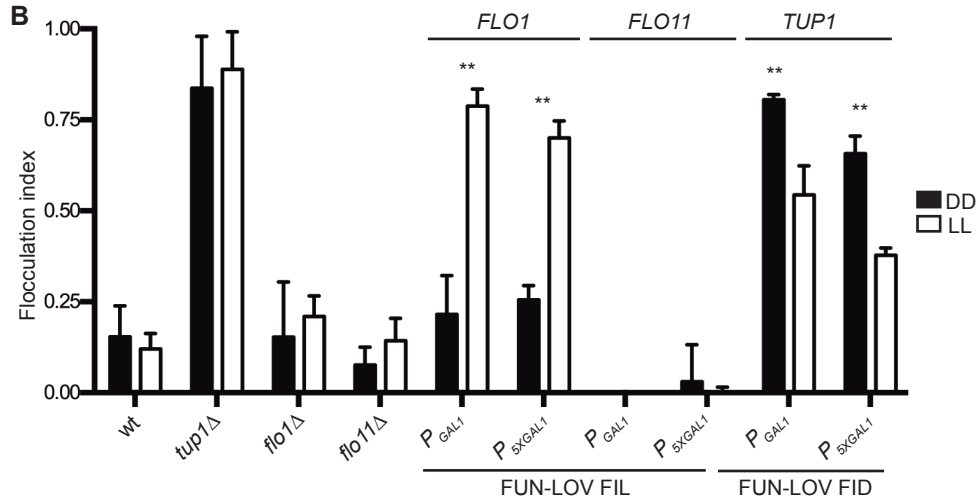

Supplement: FIG S8 [file mbo004183986sf8.pdf]
