# Supplementary material for: Fungal Light-Oxygen-Voltage Domains for Optogenetic Control of Gene Expression and Flocculation in Yeast
Source: mBio. 2018 Jul 31;9(4):e00626-18. doi: 10.1128/mBio.00626-18 (PMC6069114; doi:10.1128/mBio.00626-18)
Supplement: TABLE S1 [file mbo004183986st1.pdf]

Table S1. Primers used in this work

For plasmids assembly utilizing yeast recombinational cloning.

Name	Sequence (5'-3')	Length (bp)		Description
13	AGCGGATAACAATTTCACACAGGAA<br>ACAGCTAGGCGCATGCAACTTCTTT	50	FW	*YRP-L-*P_{ADH1}
oL3148	GGTAACGCCAGGGTTTTCCCAGTCA<br>CGACGCCGGTAGAGGTGTGGTCAAT	50	RV	*ADH2*$_{ter}$*-YRP-R*
19	AGCGGATAACAATTTCACACAGGAA<br>ACAGCATCGATGAATTCGAGCTCGT	50	FW	*YRP-L-KanMxRV*
20	CGGGTTAATTAAGGCGCGCC	20	RV	*KanMxRV*
21	AAACAGATCTGGCGCGCCTTAATTA<br>ACCCGACTAGTACGGATTAGAAGCC	50	FW	*KanMxRV-*P_{GAL1}
oL3026	aaacagatctggcgcgccttaatta<br>acccgcggaggacagtactccgctc	50	FW	*KanMxRV-*P_{5XGAL1}
oL3080	GCCCTTCTTAATGTTCTTAGCATCG<br>GCCATGGTAAGCTTAATATTCCCTA	50	RV	*Luc-*P_{GAL1}
oL3079	CGACTCACTATAGGGAATATTAAGC<br>TTACCATGGCCGATGCTAAGAACAT	50	FW	P_{GAL1}*-Luc*
oL3081	GGTAACGCCAGGGTTTTCCCAGTCA<br>CGACGTGGATCCTTGCAAATTAAAG	50	RV	*CYC1*$_{ter}$*-YRP-R*
22	GGTAACGCCAGGGTTTTCCCAGTCA<br>CGACGGGTAAGCTTAATATTCCCTA	50	RV	P_{GAL1}(P_{5XGAL1})*-YRP-R*
oL3150	AGCGGATAACAATTTCACACAGGAA<br>ACAGCATTTCAAAGAATACGTAAAT	50	FW	*YRP-L-*P_{TDH3}
oL3151	CATGTTATCCTCCTCGCCCTTGCTC<br>ACCATTTTGTTTGTTTATGTGTGTT	50	RV	*mCherry-*P_{TDH3}
oL3152	GTTTCGAATAAACACACATAAACAA<br>ACAAAATGGTGAGCAAGGGCGAGGA	50	FW	P_{TDH3}*-mCherry*
oL3153	GAATGTAAGCGTGACATAACTAATT<br>ACATGCTACTTGTACAGCTCGTCCA	50	RV	*CYC1*$_{ter}$*-mCherry*
oL3154	ACCGGCGGCATGGACGAGCTGTACA<br>AGTAGCATGTAATTAGTTATGTCAC	50	FW	*mCherry-CYC1*$_{ter}$
oL3266	TTTTGGGACGCTCGAAGGCTTTTAA<br>TTTGCcgggttaattaaggcgcgcc	50	FW	*CYC1*$_{ter}$*-HphMx*
oL3267	aaacagatctggcgcgccttaatta<br>acccgGCAAATTAAAAGCCTTCGAG	50	RV	*HphMx-CYC1*$_{ter}$
oL2562	gGTAACGCCAGGGTTTTCCCAGTCA<br>CGACGatcgatgaattcgagctcgt	50	RV	*HphMx-YRP-R*

Primers used for promoter swapping

Name	Sequence (5'-3')	Length (bp)		Description
oL3024	ctctttttcctaataaggtggagct<br>tttggcttccagtatgctttcacgg<br>atcgatgaattcgagctcgt	70	FW	$P_{FLO1\Delta}$::*KanMxRV-*<br>P_{GAL1}(P_{5XGAL1})
oL3025	gccagaagtgtaaagactgccaaaa<br>acatatagcgatgaggcattgtcat<br>ggtaagcttaatattcccta	70	RV	$P_{FLO1\Delta}$::*KanMxRV-*<br>P_{GAL1}(P_{5XGAL1})
oL3055	ATTCTCATCGAGAGCCGAGCCATAC<br>ACCTAAGGTGGACAGAAAGCTAAAA<br>ATCGATGAATTCGAGCTCGT	70	FW	$P_{FLO11\Delta}$::KanMxRV-<br>P_{GAL1}(P_{5XGAL1})
oL3056	TTAAATAGAAGCGAAAGGACCAAAT<br>AAGCGAGTAGAAATGGTCTTTGCAT	70	RV	$P_{FLO11\Delta}$::*KanMxRV-*<br>P_{GAL1}(P_{5XGAL1})

	GGTAAGCTTAATATTCCCTA			
oL3233	CTTCACTCTTGACGCCAGTTGAGTG AGGGACTGAATACTATATACAGTAA ATCGATGAATTCGAGCTCGT	70	FW	$P_{TUP1\Delta}$::*KanMxRV*-P_{GAL1}(P_{5XGAL1})
oL3261	TCGAGAAGCTCATTCAGCTTATTCT GCGTATTCGAAACGCTGGCAGTCATgg taagcttaatattcccta	70	RV	$P_{TUP1\Delta}$::*KanMxRV*-P_{GAL1}(P_{5XGAL1})

Primers used promoter-swapping confirmations.

Name	Sequence (5'-3')	Length (bp)		Description
oL2789	ttggtaacgatgagggaacc	20	FW	Upstream *FLO1*
oL3050	ACTTTTCCTCTGGCCTGCTG	20	RV	Internal *FLO1*
oL3163	TACCGTCGTTTTGGGGTTCC	20	FW	Upstream *FLO11*
oL3071	TTGGGACAGCCATTAACGAT	20	RV	Internal *FLO11*
oL3262	GCGGAATCGATCTGTTGTTT	20	FW	Upstream *TUP1*
oL3263	CTTCTTCGTACGCGTCCTTC	20	RV	Internal *TUP1*
21	AAACAGATCTGGCGCGCCTTAATTA ACCCGACTAGTACGGATTAGAAGCC	50	FW	*KanMxRV*-P_{GAL1}
oL3026	aaacagatctggcgcgccttaatta acccgcggaggacagtactccgctc	50	FW	*KanMxRV*-P_{5XGAL1}
oL2091	CATCCTATGGAACTGCCTCGG	21	FW	Internal *KanMx*

Primers used for gene deletion and reporter gene integration in the genome.

Name	Sequence (5'-3')	Length (bp)		Description
oL2787	Ctctttttcctaataaggtggagct tttggcttccagtatgctttcacgg cggcatcagagcagattgtactg	73	FW	*flo1*Δ::*URA3*
oL2806	Tactcgaaaacatcctaagcgaacc acactagatcttacgttagtactgc ggtatttcacaccgcataggg	71	RV	*flo1*Δ::*URA3*
oL3072	TGCGGTATCTTCACGGACAGAACTT CTATTGCCTATCGGTGGTGTGATTA cggcatcagagcagattgtactg	73	FW	*flo11*Δ::*URA3*
oL3073	CGAAGATTATTAGTTGTGCCAAGGC AATATCAGGTTTATTAATCTTTTAG ggtatttcacaccgcataggg	71	RV	*flo11*Δ::*URA3*
oL2120	CCAGCGTATACAATCTCGATAGTTG GTTTCCCGTTCTTTCCACTCCCGTC CGGGTTAATTAAGGCGCGCC	70	FW	*gal80*Δ::*HphMx*
oL2121	GTTTTTATAACGTTCGCTGCACTGG GGGCCAAGCACAGGGCAAGATGCTT ATCGATGAATTCGAGCTCGT	70	RV	*gal80*Δ::*HphMx*
oL2122	ATGCACAGTTGAAGTGAACTTGCGG GGTTTTTCAGTATCTACGATTCATT CGGGTTAATTAAGGCGCGCC	70	FW	*gal4*Δ::*NatMx*
oL2123	AATGCACGCCATCATTTTAAGAGAG GACAGAGAAGCAAGCCTCCTGAAAG	70	RV	*gal4*Δ::*NatMx*

oL3082	ATCGATGAATTCGAGCTCGT AGGAGTGCAAAAAGAGAAAATAAAA GTAAAAAGGTAGGGCAACACATAGT	70	FW	*gal3*Δ::*KanMxRV*-P_{GAL1}(P_{5XGAL1})-*Luc*
oL3083	atcgatgaattcgagctcgt TATGAGTAAACTTTTAATATTTAAA GGTTGTTCCAAGAAGGTGTTTAGTG TGGATCCTTGCAAATTAAAG	70	RV	*gal3*Δ::*KanMxRV*-P_{GAL1}(P_{5XGAL1})-*Luc*

Primers used for confirmation of gene deletions and genome integration.

Name	Sequence (5'-3')	Length (bp)		Description
oL2789	ttggtaacgatgagggaacc	20	FW	Upstream *FLO1*
oL2289	CCTCTAGGTTCCTTTGTTACTTCT	24	RV	Internal *URA3*
oL2807	tcacctgctttgaatcgttg	20	RV	Upstream *FLO1*
oL2288	CCTTTTGATGTTAGCAGAATTGTC	24	FW	Internal *URA3*
oL2099	GAGACAGCATTCGCCCAGTA	20	FW	Upstream *GAL4*
oL2163	GATTCGTCGTCCGATTCGTC	20	RV	Internal *NatMx*
4	AGGTTACATGGCCAAGATTGA	21	RV	Upstream *GAL4*
oL2164	AGGTCACCAACGTCAACGCA	20	FW	Internal *NatMx*
oL2096	TCTTCATTTACCGGCGCACT	20	FW	Upstream *GAL80*
oL2094	CGGCGGGAGATGCAATAGG	19	RV	Internal *HphMx*
oL2097	CGCTGCTGCAAAGTTTTGAC	20	RV	Upstream *GAL80*
oL2095	TCGCCCGCAGAAGCGCGGCC	20	FW	Internal *HphMx*
oL3078	CAGTCCGAGCGTTTAGAAGG	20	FW	Upstream *FLO11*
oL3089	ATGAAATCGCCATGCCAAGC	20	FW	Upstream *GAL3*
oL2091	CATCCTATGGAACTGCCTCGG	21	FW	Internal *KanMx*
oL3127	GTGCGGAGCCACTCTGACTC	20	RV	Upstream *GAL3*
oL1734	GGCCGGCCGCTTCGAGCAGACATGA TAAGAtcatgtaattagttatgtca	50	FW	*CYC1*$_{ter}$-*Luc*

Primers used for real time PCR (qPCR).

Name	Sequence (5'-3')	Length (bp)		Description
oL3682	GGGTTTGCGTTCTGTGATCT	20	FW	*HEM2 housekeeping*
oL3683	TTGAATGACTGGCCCTGCAG	20	RV	*HEM2 housekeeping*
oL3692	GGATCAGGTCTTCCGTAGCG	20	FW	*TAF10 housekeeping*
oL3693	TGAAATCTGCTGCACGCCA	19	RV	*TAF10 housekeeping*

oL3321	TGCCGTCACAGATAGATTGG	20	RV	*GAL4-BD*
oL3322	CTCTTCCGATGATGATGTCG	20	FW	*GAL4-BD*
